# Haematuria and Office Urology
*A paper read to the Surgical Club of South West England, 26th April 1963.


**Published:** 1963-10

**Authors:** Hugh Guerrier


### HAEMATURIA AND OFFICE UROLOGY*

BY

HUGH GUERRIER

At a recent meeting of the Urological Section of the Royal Society of Medicine, it was stated that 25 per cent of all the work done by general surgeons was urology; there is, therefore, an excuse for addressing you on two aspects of the subject which may be of some interest.

### HAEMATURIA

First, I would like to say a word about the symptom of haematuria, a topic on which little has been written. Indeed, I have only been able to find two articles in the British literature, one by Debenham in the British Journal of Surgery for 1933 and one by Riches in a Presidential Address to the Medical Society of London, 21 years later. It is interesting to note that in the study of Debenham's cases, excretion pyelography was not used, because it had not then been invented.

Haematuria is, of course, an extremely important symptom for several reasons. First, it is very alarming to the patient, who in consequence tends to seek advice early, and therefore failure to investigate it early is more often due to the doctor's shortcomings than to those of the patient. Secondly, of all the various symptoms of which a patient may complain and which may be difficult to localise, haematuria at least fixes the urinary tract as the source of the symptoms. Thirdly, and I think this is important, although haematuria is a very common symptom to a surgeon, it is certainly not so to a general practitioner.

My observations are based on a personal consecutive series of 528 cases seen in 8½ years, that is an average of 63 cases per annum, so that one is constantly engaged in investigating this symptom. It appears that this is a reasonable number of cases to discuss and compares not unfavourably with Riches' 1000 cases seen in 12 years and Debenham's 742 cases in 7 years at the London Hospital.

But these 528 cases of mine were sent to me by 105 general practitioners, hence on average each practitioner referred only 5 cases in 8 years and must, therefore, regard the symptom as uncommon. Moreover, if the haematuria is due to an inflammatory condition which subsides either spontaneously or as the result of simple treatment, the rarity combined with the often permanent remission induces in the minds of many the notion that haematuria is an unimportant symptom. It is certainly my experience that many patients are only sent to me after 2 or 3, or even 4 or more episodes of haematuria, sometimes spread over many months or even years. It is unnecessary for me to mention the serious lesions which may give rise to this symptom, but it is true that many of them may be permanently cured if treated promptly, whilst investigation of others may put the patient's mind at rest at an early stage.

The amount of blood lost is not generally of serious consequence, but severe haemorrhage may occur, and some years ago I had a patient literally pouring blood from his urethra. His haemoglobin had dropped to 20 per cent and twelve pints of blood were required to combat his state of shock. Diagnosis was a problem, he was far too ill for radiography, and cystoscopy was hopeless for even after repeated bladder washouts with an evacuator his rate of bleeding gained on us; but cystotomy disclosed

* A paper read to the Surgical Club of South West England, 26th April 1963.

a gigantic papilliferous tumour, perfectly benign. I think it is true to say that, in the absence of trauma, a really catastrophic haematuria is most likely to arise from a bladder lesion.

What about the diagnosis of haematuria? Please don't think that I am about to insult you by telling you how to diagnose the cause of this symptom, but there are, I think, one or two points worth remembering. First, it is necessary to make sure that one really is dealing with haematuria.

Women can be very confusing and confused about the site of what they believe is blood in the urine; the urethra and vagina and even the anus may be the origin of the flux, whilst men may be sent labelled haematuria with an obvious history of haemospermia.

Again, is the fluid really blood? Haemoglobin, myoglobin and melanin may all appear in the urine on rare occasions, and so from time to time may anthocyanin (a pigment which colours the urine red) from the excessive consumption of beetroot. This last occurred in one of my own children who got loose in the larder, and whose red urine caused temporary dispondancy until the pigment was isolated. Often, of course, one must rely on the history, for many cases of haematuria have a habit of temporary spontaneous remission and a specimen containing pigment is often not available.

Having established that true haematuria is the symptom, what are its causes? These are, of course, general and local, the latter being confined to the urinary tract itself.

As surgeons we are used to dealing with localized conditions, but I think perhaps it is not inappropriate to mention the general causes:—

First—Acute glomerulonephritis, commonest in children and associated with oedema, hypertension and albuminuria, in addition to haematuria. Granular casts are present in the urine and there is a raised blood urea. The condition always follows some streptococcal infection, commonly scarlet fever, and there is a rise in the anti-streptolysin titre.

Secondly—Focal nephritis is a condition in which changes occur in the afferent glomerular arteriole, the nature of which is not clear, but which are associated with granular casts in the urine and haematuria. However, there is no oedema, no hypertension and no azotoemia. The condition appears to have a favourable prognosis and can only be diagnosed with certainty by renal biopsy.

Thirdly—Embolic phenomena, from lesions such as auricular fibrillation or bacterial endocarditis.

Fourthly—Blood dyscrasias, leukaemia, haemophilia, thrombocytopenic purpura.

Fifthly—Iatrogenic haematuria. This is becoming increasingly common because of long-term anti-coagulant therapy.

Finally—Haematuria may occur following strenuous exercise such as cross-country running. It occurs most commonly in children and adolescents. This is a true haematuria and not due to haemoglobin or myoglobin. It appears not to be of serious significance and disappears spontaneously as age advances. Its importance lies in its distinction from haematuria due to organic disease.

With regard to local lesions, everyone knows that the classical five of Trauma, Tumour, Tubercle, Inflammation and Stone account for the majority of cases. In my own series they accounted for 87 per cent of all completely diagnosed cases.

On examination, physical signs are only elicited in the minority of patients, except for the findihg of a distended bladder or a lesion in the prostate.

Digital examination of the prostate per rectum is remarkable for its accuracy and the bimanual examination of prostate and bladder and the rest of the pelvis under full relaxation is invaluable. It can be performed at the time of the cystoscopy and should never be omitted.

Concerning investigation, I think that all cases require intravenous pyelography as well as cystoscopy, in addition, of course, to full examination of the urine.

Little help, however, is obtainable from the blood chemistry. The blood urea is most important as an indication of renal function, but does not help much with diagnosis. The serum acid phosphatase is only positive in carcinoma when secondaries are present and generally only when they are in bone. A normal value is, therefore, of no significance. It may be noted, however, that a paper was read at the section of Urology on 28th March this year, indicating that the total serum acid phosphatase may be divided into fractions derived from various organs and that the "Prostatic" or tartrate labile fraction is frequently raised above its normal value of 0.8 King Armstrong units in prostatic carcinoma in which the lesion appears to be confined to the gland, so that in the future this investigation may prove of greatly increased diagnostic value. The serum calcium and phosphorus may assist, in very rare occasions, in demonstrating hyperparathyroidism as a cause of stone or nephrocalcinosis.

The value of excretion pyelography has, of recent years, been enormously increased by renal tomography and I have been very fortunate in that Dr. Robinson Thomas started this method at Newton Abbot Hospital some ten or twelve years ago. The criticism that many serial cuts at different levels exposes the patient to excessive irradiation is met by the newer techniques in which three cuts at different levels are taken on one exposure and hence six different levels can be taken with only one additional dose. Accurate pictures are now thus obtained in spite of the gas or obesity which hitherto has rendered renal radiography almost useless in the average Devonian. I prefer to have an excretion pyelogram done before cystoscopy, for if the films are unsatisfactory for any reason, a retrograde pyelography may be performed at the time of the first endoscopy and thus save time and an additional endoscopy for the patient.

Endoscopy is essential in all cases of haematuria, except perhaps in obvious acute nephritis. Clearly the diagnosis may be made by endoscopy alone, but haematuria may be associated with lesions in kidney or ureter, as well as bladder, so that pyelography and endoscopy should be performed in every case of haematuria; moreover, endoscopy permits of the biopsy of lesions, etither by using biopsy forceps or better still, with the resectoscope.

The only remaining method of investigation is by renal angiography. At this hospital we are making prelininary studies in this field, but as yet I am not in a position to comment from a personal series.

The important thing, of course, is how successful are one's attempts to elucidate the cause of haematuria.

In my own series there were 528 patients, many of whom had long since stopped bleeding, and were without symptoms when first seen at my clinic. The diagnosis was complete in 428, or 81 per cent. Of the remaining 100 or 19 per cent, 34 left the district or for other reasons did not present themselves for full investigation, but 66 or 13 per cent of the total revealed no cause for haematuria after the fullest investigation. Although the ratio of men to women in the whole series was 2.5: 1, in this last group there were 39 undiagnosed men and 27 undiagnosed women, that is 11 per cent of the total number of men were undiagnosed, but 20 per cent of the total number of women were, almost twice the percentage. It is my belief that this is due to the frequency, in women, of haematuria caused by urinary infection which completely subsides before investigation can be begun, and this accounts, in some measure, for the depressingly high total figure of 13 per cent in which there was failure to find a cause.

The question is, how many lesions of serious consequence were missed; what horrors lay hidden in this 13 per cent? Follow-up questionnaires were sent to all the patients. Of the 66 who failed to reveal any lesion 40 replied. Of these, 32 had been symptomfree for a period of two to seven years; and of the remaining eight, one has died of pneumonia and seven have been followed from 2–7 years with an occasional inflammatory episode or rare haematuria, but all have remained well.

Although I cannot claim that every patient with haematuria has been followed to the end, I think that this investigation tends to show that if all cases of haematuria are fully investigated by all the means at our disposal, very few lesions of serious significance are likely to escape detection.

### OFFICE UROLOGY

This subject of investigation brings me to the second topic about which I should like to say something, namely, what I believe is known in the States as Office Urology, that is the performance of urological investigation and treatment, if not in the consulting room, at least as out-patient procedures. I am well aware that such procedures are commonplace in the teaching centres and larger hospitals, but I believe that there may be an even greater need for such facilities in the smaller hospitals, dictated largely by the ever pressing demand for beds.

"More beds" is the cry on every surgeon's lips, but in popular holiday resorts such as Torquay the bed situation is serious indeed. I really won't weary you with civic statistics, but when I tell you that the basic population of Torquay alone is 50,000, that during August it is a quarter of a million with the influx of 200,000 visitors, and that from May to September it averages about 200,000 you will see that the potential for clinical material is considerable, and this takes no account of Paignton, Newton Abbot nor the rest of the area we serve. When in addition you realize that a large number of the hotels are owned by North Country Motor Coach Companies, whose vehicles are filled with the elderly and with crates of beer, so that their predetermined and infrequent stops result, on arrival, in immediate admission to hospital, you will appreciate that our beds are at a premium.

This situation has resulted in considerable delays; urological investigation has suffered heavily and, on occasion, cases of haematuria with an equivocal I.V.P. have been put down to come into hospital for retrograde examination, which, when performed twelve months later, has revealed a growth now inoperable. As for papilloma follow-ups, their admission remained a pious hope and thus the need to avoid in-patient admissions became paramount. This the more compelling with anyone who, like myself, takes a particular interest in urology.

Urology forms at least 60 per cent of my work, perhaps more, and I became convinced that an endoscopy clinic, completely closed, forming an extension of my own general surgical out-patient clinic and not in any way an official hospital clinic, was essential if my patients were to have any chance of being properly investigated and, in many cases, treated.

I felt that some of you who may feel the need of similar facilities might possibly be interested in these requirements and how they were met in a small peripheral hospital. These requirements may be considered under six headings:—

1. Accommodation.
2. Equipment.
3. Medical staff.
4. Nursing staff.
5. Radiographic staff.
6. Transport.

*Accommodation*. The minimum needs are a room for the patient to wait in, and suitably screened in which to recover; and a room in which to work, that is, to anaesthetise the patient, perform the various endoscopic manoeuvres, take X-rays and so on.

In addition to this an adjoining dark room is most important and so is somewhere to lay trolleys with sterile instruments.

I have, I think, been very fortunate in this, for with the invaluable co-operation of our Radiologist I use an old X-ray room, partly vacated when the new department was built. It is equipped with a modern tube and adjacent dark room, scrubbing up facilities and even a telephone, and on the opposite side of the corridor is a room for patients to recover.

*Equipment.* The first essential is a suitable table. This may be an expensive item for which limited hospital funds are inadequate. I have a Hugh Young table specifically designed for urological work and fitted with a Potter-Bucky diaphragm. A number of these tables arrived in this country during the War under the lease-lend agreements and there are still some available from the Ministry of Supply.

I was lucky in catching the Regional Board in an unguarded moment and obtained a special grant from it. This, plus an introduction to a woman in the Ministry's office in Tavistock Square resulted in her piloting me to a dump at the back of Cricklewood where a number of these, each unused and fitted with a small X-ray tube, were available.

The one I chose was sent down and our Works Department removed the tube and fitted wheels and brakes, so that the table could be run alongside a modern X-ray machine. The Radiologist was kind enough to have his tube modified so that it would swing through 180° and thus serve my table as well as his own. Finally, the hand operated Bucky was converted to electrical operation.

The instruments must be a complete set, cystoscope with diagnostic and operating telescopes, panendoscope, endoscopic biopsy forceps, resectoscope and evacuator, ureteric catheters and ureterotomes, bougies of all kinds, reservoir and suitable trays for sterilising instruments in hibitane.

*Medical Staff.* In addition to the surgeon one must have an anaesthetist who can induce unconsciousness quickly, keep the patient that way for anything from two minutes to three-quarters of an hour or even more at a moment's notice, and then have the patient sufficiently conscious to be taken home later in the afternoon.

*The Nursing Staff* needs are critical. I don't really think there is any place for untrained staff in such a unit. It takes a while to get even trained nurses used to the hurly-burly of the business and each girl must be able to carry out her own particular job without supervision.

I have a staff nurse who runs the clinic, makes all the appointments and generally arranges matters, so that we have men one afternoon and women the next. She also looks after the anaesthetist. She is ably assisted by another staff nurse who acts as organising deputy and looks after the surgeon and his instruments, lays the trolleys and so on. In addition one needs a third staff nurse to supervise waiting patients, and to take care of those regaining consciousness. Finally, a male nurse to give such local anaesthesia to male patients as is required, lift and transport patients, and act as general factotum. So that we need a minimum of three nurses and one male nurse, and if on occasions we can get a fourth we always seem to keep her busy.

With regard to *Radiographic staff*, the Radiologist kindly provides one of his staff to take the pictures, whilst Dr. Holden himself usually comes to give his blessing and discuss any difficult problems as they arise.

Finally *Transport.* All the patients are taken home, occasionally in a slightly muzzy condition, by the Hospital Car Service, without whose help I doubt if we could function.

The type of work done is as follows: Diagnostic cystoscopy and panendoscopy, biopsy, endoscopic ureterotomy for stones low in the ureter, crushing and evacuation of stones in selected cases and limited bladder neck resections in chronic cystitis in females. Retrograde pyelograms and ureterograms form an important part of the work and I believe can only be satisfactorily undertaken with this kind of unit. In many hospitals, including my own, the X-ray department is far from the theatre, and the only means of doing a retrograde pyelogram is by passing the ureteric catheter in the theatre and then, having withdrawn the cystoscope, the patient is wheeled to the X-ray department, during which time almost anything can happen to the ureteric catheter.

Again, fine adjustments of the catheter cannot be accurately performed unless the cystoscope is in place. In some hospitals there are facilities for cystoscoping patients in the X-ray department, but often only on a standard X-ray table and only in the supine position. With the facilities which I have described, the patient may be cystoscoped either supine or in the lithotomy position, he is not moved for his X-rays, the table can be put into quite a steep Trendelenberg position if required, and the catheter can be adjusted as often as may be necessary.

The most important limiting factor in a unit like this is the space available for patients to recover. It is astonishing how much room a few unconscious and semi-conscious bodies take up, all, of course, requiring supervision.

On several occasions I have done ten cases in an afternoon; this may not seem a very large number to some of you, but it strains our resources to the limit and six or seven are more satisfactory. At the moment we are doing about 250 a year and the clinic has been working for exactly three years today, so I feel we have saved a worth-while number of beds.

Some may ask if it is safe to do this kind of work on out-patients. I think it is justifiable. Of the approximately 750 cases that have passed through the clinic, two were admitted to hospital forthwith because of post-instrumental bleeding which was judged too severe to allow of their returning home, and two, both of whom had strictures, were admitted the following day for difficulty in micturition. None of these patients came to any harm and all were discharged in a few days in a satisfactory condition.

What are the advantages of such a clinic? First it is possible more or less to keep pace with the numbers requiring investigation and to be able to give the G.P's a complete answer to a urological problem in a relatively short time.

The patients are first seen with the general surgical cases in my ordinary out-patient clinic. Having had the routine there, an I.V.P. is ordered and they are put on the waiting list for the Endoscopy clinic the Wednesday following their I.V.P. If I should see in my general clinic on a Tuesday a really urgent case, he can be cystoscoped, if necessary, the following afternoon. Secondly, in addition to investigation, conditions such as papillomata, certain cases of stone in bladder or lower ureter, chronic cystitis in females with bladder neck obstruction and so on, can be treated without hospital admission.

Thirdly, surprising as it may seem, the patients prefer it, in spite of the rough and tumble of my slightly primitive set up. Very few patients want to be admitted and all the papilloma checks and other cases who require regular follow-up, turn up regularly and say they prefer it that way. Those at work are only away from work for an afternoon and mothers don't have to leave their families for a night or more.

Fourthly it is a splendid opportunity to teach one's house surgeon to use a cystoscope. So often a cystoscopy is put on the end of an ordinary operating list and the houseman allowed only a brief glimpse because the list has been long and everyone is tired. He may, but generally doesn't, see the ureteric orifices, and learns very little. But even in this small set-up of mine he may see 100 or 150 cystoscopies in six months. He handles the instruments, describes what he sees and is then checked by the surgeon, and by the end of six months he is quite handy with a cystoscope and won't be, as I was, in terrified ignorance of endoscopic appearances and procedures until I had been a urological registrar for some time.

Finally, we all like it; the nurses because it is different, the anaesthetist because the sudden variations in length of procedure and the necessity for a rapid return to consciousness tests his skill, and I like it for one sees a lot of very interesting material in a short time, and it is really great fun.

In conclusion, I should like to pay tribute to the absolutely splendid co-operation I have had from the nursing staff whose constant care and happy demeanour have done so much to give the patients confidence, without which we could never persuade them to come.

